# Continuous bioreactor production of polyhydroxyalkanoates in *Haloferax mediterranei*

**DOI:** 10.3389/fbioe.2023.1220271

**Published:** 2023-09-13

**Authors:** Mariana Parroquin-Gonzalez, James Winterburn

**Affiliations:** Department of Chemical Engineering University of Manchester, Manchester, United Kingdom

**Keywords:** bioreactor, fermentation, continuous, *Haloferax mediterranei*, polyhydroxyalkanoates, Poly(3-hydroxybutryrate-co-3-hydroxyvalerate)

## Abstract

In this work, the viability of continuous poly(3-hydroxybutyrate-co-3-hydroxyvalerate) (PHBV) production with controlled composition in *Haloferax mediterranei* when fed volatile fatty acids is demonstrated. Continuous fermentations showed to greatly outperform batch fermentations with continuous feeding. Operating the bioreactor continuously allowed for PHBV productivity normalised by cell density to increase from 0.29 to 0.38 mg L^−1^ h^−1^, in previous continuously fed-fed batch fermentations, to 0.87 and 1.43 mg L^−1^ h^−1^ in a continuous mode of operation for 0.1 and 0.25 M carbon concentrations in the media respectively. Continuous bioreactor experiments were carried out for 100 h, maintaining control over the copolymer composition at around 30 mol% 3-hydroxyvalerate 3HV. This work presents the first continuous production of PHBV in *Haloferax mediterranei* which continuously delivers polymer at a higher productivity, compared to fed-batch modes of operation. Operating bioreactors continuously whilst maintaining control over copolymer composition brings new processing opportunities for increasing biopolymer production capacity, a crucial step towards the wider industrialisation of polyhydroxyalkanoates (PHAs).

## Graphical Abstract

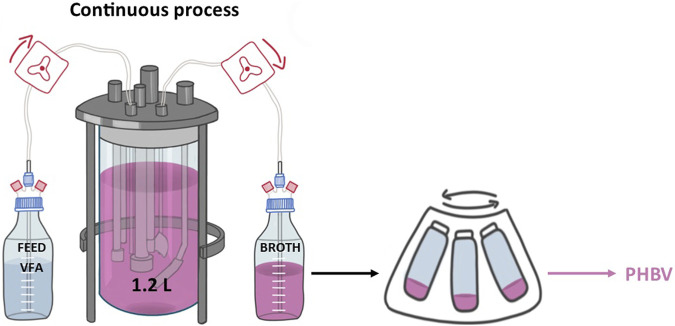

## 1 Introduction

With the high demand for plastics production worldwide and its associated processing and disposal problems there is an increasing interest in developing more environmentally friendly alternatives, including biodegradable plastics produced from a renewable source (Plastics-Europe, 2021; European Commision, 2022). Polyhydroxyalkanoates (PHAs) are a family of bioplastics that can be produced by a large range of microorganisms and possess similar mechanical properties to widely used plastics such as polypropylene and polyethylene (Doi et al., 1989; Renard et al., 2004; Platt, 2006; Khanna and Srivastava, 2008). The homopolymer poly-3-hydroxybutyrate (PHB) is one of the most commonly produced PHAs. However, when adding the 3-hydroxyvalerate (3HV) monomer into the chain to form the copolymer poly-3-hydroxybutyrate-co-3-hydroxyvalerate (PHBV) the high crystallinity of the PHB is disrupted and a more malleable and flexible polymer is created which presents suitability for a broader range of industrial applications (Anjum et al., 2016).

The main restrictions on PHA industrial scale-up lies on their relatively high production cost, with the estimated PHA market price ranging between 2.4 and 5.5 USD per kg while the petroleum-based alternatives cost around 1 USD per kg (Gholami et al., 2016; Crutchik et al., 2020). A large proportion of the costs are associated with creating sterile conditions, substrate carbon source refinement, use and disposal of solvents for polymer extraction and the low productivity, yields and other limitations such as short production campaigns and downtime for batch changeover of batch/fed-batch modes of operation (Arcos-Hernández et al., 2013; Kumar et al., 2016; Kourmentza et al., 2017; Chen and Jiang, 2018). Halophilic microorganisms have been suggested as suitable PHAs producers at a lower financial cost; the high salinity of the production medium needed (150–200 g/L of salts) for halophiles to grow minimises the risk of contamination, removing the need for sterile conditions and facilitates operation allowing for open vessels and low contamination risk continuous process (Chen and Jiang, 2018; Mahler et al., 2018; Kasirajan and Maupin-Furlow, 2021). Furthermore, the downstream process can be hugely simplified, extracting the polymer by simple osmotic shock when the cells are transferred into an isotonic media, removing the need for toxic and expensive solvents (Oren, 2010; Koller et al., 2017).

In most microorganisms, the 3HV percentage in PHBV is limited by the precursor toxicity with compositions above 50% being hard to obtain (McChalicher and Srienc, 2007; Han et al., 2015). However, *Haloferax mediterranei* can produce PHBV naturally without the need of precursors and can utilise a wide range of cheap carbon sources as substrate making it one of the preferred halophilic archaea for PHA production (Chen et al., 2006; Huang et al., 2006; Koller et al., 2007; Pais et al., 2016; Alsafadi and Al-Mashaqbeh, 2017; Montemurro et al., 2022). In addition, polymer composition could vary from batch to batch delivering a product with fluctuating mechanical properties. It has been shown that by feeding a mix of C4:0 and C5:0 volatile fatty acids (VFAs) the polymer composition can be controlled; in a fed-batch fermentation the 3HV content is directly proportional to the fraction of C5:0 fed (Ferre-Guell and Winterburn, 2018).

With sterilisation, extraction and carbon substrate simplified, the next productivity improvement can come from the operation mode selected. In addition to the fermentation time itself, batch operations include long periods of downtime, during which equipment is cleaned and sterilised, media is prepared, and a new inoculum is grown. This contributes to delayed product delivery or the need to set up several batches in series which complicates the process and requires more capital investment or result in less overall production from a given facility. Continuous fermentations present an attractive alternative with an ease to control the fermentation allowing for a constant polymer harvest with a single longer production campaign.

A semi-continuous, fed batch, mode of operation has previously been tested, where the fermentation was continuously fed a mix of C4:0/C5:0 VFA in order to keep the concentration in the media constant, without an outlet stream (Parroquin Gonzalez and Winterburn, 2022). This strategy led to a productivity increase (12.8 mg L^-1^ h^-1^) when compared to a pulse-feeding fed batch strategy (3.4 mg L^-1^ h^-1^) (Ferre-Guell and Winterburn, 2018; Parroquin Gonzalez and Winterburn, 2022). However, the amount of polymer produced over time in the continuously fed fed-batch fermentation (CFFB) was limited by the reactor volume, increased cell density over time and accumulation of other metabolites and by-products, in addition the downstream process associated to a batch operation was still present. It has been shown that fully continuous operation can further increase the process productivity and simplify the operation generating a more industrially attractive process (Garcia Lillo and Rodriguez-Valera, 1990; Chen and Jiang, 2018; Amstutz et al., 2019). Therefore, a fully continuous fermentation is the crucial next step in improving polymer production. This has already been implemented for other PHA producing species showing promising results, including the genetically engineered *Halomonas TD01* (Yu et al., 2005) and *Ralstonia eutropha* (Fu et al., 2014). *Haloferax mediterranei* and the process described here present an opportunity for a non-engineered species that can operate under non-sterile conditions.

When performing a continuous fermentation, the genomic stability of the culture might be at risk. There are some halobacteria known to have high genomic stability. In 1990, Garcia Lillo & Rodriguez-Valera performed a continuous fermentation with *Haloferax mediterranei* for 3 months at a dilution rate of 0.12 h^-1^ which roughly corresponds to 370 generations. Afterwards they tested the PHB production of the strain and did not observe any significant difference from the original strain deeming *Haloferax mediterranei* as a stable culture for continuous operations (Garcia Lillo and Rodriguez-Valera, 1990).

The work presented in this paper details the first effort to continuously produce PHBV using *Haloferax mediterranei* in a continuous mode of bioreactor operation with VFAs as substrate, with the aim of further improving process productivity. Bioreactors were continuously fed a mix of C4:0/C5:0 VFAs maintaining steady state carbon concentration, cell density and biopolymer concentration in the media, proving the viability of the process. Following this continuous bioreactor operation strategy improved both productivity normalised by optical density (OD) and yields, presenting a process that has the potential to be more industrially attractive. This work represents a crucial step for the wider industrialisation of biopolymer production.

## 2 Materials and methods

### 2.1 Microorganism and fermentation conditions

*Haloferax mediterranei* (DSM 1411, ATCC 33500) obtained from the Leibniz Institute DSMZ-German Collection of Microorganisms and Cell cultures, was used for all experiments. Cryovials of working stock were stored at −80°C. The seed culture was initiated by reviving the cryovial content in 20 mL minimal synthetic media (MSM) supplemented with 10 g/L of glucose and incubated during 24 h at 37°C and 200 rpm (INNOVA 42, New Brunswick Scientific). This was then transferred to a 1 L shake flask with 200 mL MSM supplemented with 10 g/L of glucose and incubated for further 36 h at 37°C and 200 rpm. Prior to starting the fermentations, cells were centrifuged to remove all media supplemented with glucose. For a final cultivation phase, the cells were resuspended in 200 mL MSM with 0.1 M carbon concentration of C4:0/C5:0 mix with a 50/50 ratio and inoculated for 48 h. This final cultivation phase was designed to adjust the cells to the fatty acids and reduce the lag phase at the beginning of the fermentation in the reactor. Finally, the cells were centrifuged at 7,000 rpm for 10 min and the supernatant was discarded. The pellet was then resuspended in fermentation media and added to the corresponding bio reactor.

The composition of the minimal synthetic media (MSM) was (g/L): 156.0 NaCl, 13.0 MgCl_2_ · 6H_2_O, 20.0 MgSO_4_ · 7H_2_O, 1.0 CaCl_2_ · 6H_2_O, 4.0 KCl, 0.2 NaHCO_3_ and 0.5 NaBr as marine salts; 0.5 KH_2_PO_4_, 0.005 (g/L) FeCl_3_ and 1.0 (mL/L) trace elements solution SL6 (10×) as supplements; 2.0 NH_4_Cl to supply the desired amount of nitrogen and 49.6 (mL/L) 1,4-piperazinediethane-sulfonic acid (PIPES) as pH buffer. The trace elements solution contained (g/L): 2.0 ZnSO_4_ · 7H_2_O, 0.6 MnCl_2_ · 4H_2_O, 6.0 H_3_BO_3_, 4.0 CoCl_2_ · 6H_2_O, 0.2 CuCl_2_ · 2H_2_O, 0.22 NiCl_2_, 0.6 Na_2_MoO_4_ · 2H_2_O. In all cases, the pH of the media was adjusted to 6.8 with NaOH or HCl solutions and with no sterilisation prior to use.

Butyric (C4:0) and pentanoic (C5:0) acid with a ≥99% purity from Sigma-Aldrich were used in all fermentations.

### 2.2 PHA bioreactor fermentations

A 3-L cylindrical bioreactor (Applikon Biotechnology, 1 Rushton turbine, 3 baffles, 2.2 H/D ratio) with an initial working volume of 1.2 L was used for all fermentations. The temperature was maintained at 37°C, the pH kept at 6.8 using 3M HCl solution, air was delivered at 0.75 vvm and an agitation cascade with stirring speed between 200 and 800 rpm was set up to maintain the dissolved oxygen at 20% [26]. C4:0/C5:0 (56:44 mol%) VFAs were used as the carbon substrate, the media had the corresponding amounts added to constitute the 0.1 and 0.25 M working concentrations.

Fermentations were continuously fed MSM media supplemented with a mix of C4:0/C5:0 (56:44 mol%) of 0.5 and 0.4 M respectively for the 0.1 and 025 M working concentration respectively. Both inlet and outlet streams were pumped with a Watson Marlow (IP31) pump with flowrates adjusted accordingly to the fermentation specifications. Samples for analytical measurements were taken from the outlet stream. No significant salt deposits were observed inside the tubing for the duration of the fermentations. Outlet broth was stored in a cold-room prior to polymer extraction.

### 2.3 Downstream: PHBV extraction

PHA from *Haloferax mediterranei* can be isolated by osmotic shock. To extract the polymer for quantification and composition analysis, 2 mL of sample broth was centrifuged in an Eppendorf tube at 13,000 rpm for 5 min and the supernatant was discarded. The pellet was resuspended in 1 mL of 0.1% w/v Sodium Dodecyl Sulphate (SDS) (Sigma Aldrich) and vortexed for 1 min. This process was repeated 3 times to eliminate the light pink colour associated with *Haloferax mediterranei* fermentation broth. Finally, cells were washed one last time with distilled water and left to dry in a drying oven at 60°C. No evidence of PHBV degradation was observed, with no discernible difference in concentration detected using GC between polymer extracted immediately after sampling and that obtained from samples stored in a cold room for a period of time prior to extraction as shown in Figure 2.

### 2.4 Analytical techniques

#### 2.4.1 Determination of optical density

Optical density was used to measure cell growth as specified in (Parroquin Gonzalez and Winterburn, 2022). 1 mL samples of fermentation broth were taken every 24 h. The sample was centrifuged for 5 min at 13,000 rpm, the supernatant retained for HPLC analysis, and the cells resuspended in a 10 w/v NaCl solution. The measurements were carried out by a US-visible spectrometer at 600 nm (UV mini-1240, UV-vis spectrometer, Shimadzu).

#### 2.4.2 Dry cell weight (DCW) and biomass

Due to the high salinity of the media, dry cell weight could not be determined by standard solvent evaporation. 10 mL of sample were analysed following the procedure detailed by (Parroquin Gonzalez and Winterburn, 2022). Samples were centrifuged for 10 min at 7000 rpm and supernatant discarded. The pellet was then resuspended in 10% w/v NaCl solution. This process was repeated three times before transferring the samples to a ceramic crucible. Samples were then dried to constant weight, weighed and then all organic matter burnt at 400°C for 4 h in a P300 furnace (Nabertherm). This process degrades all organic matter leaving the salts in the crucible. The dry cell weight was calculated as the difference between the dry weight (salts plus organic matter) and the burnt sample (salts only). Biomass was calculated as the difference between DCW and PHA concentration.

#### 2.4.3 PHA quantification and composition analysis with GC-FID

For sample preparation the method described by (Braunegg et al., 1978) was followed. 2 mL of sample were centrifuged, and the supernatant discarded. The pellet was then washed 3 times with 0.1% w/v sodium dodecyl sulphate (SDS) (Sigma Aldrich) solution before a final wash with distilled water. The pellet was then transferred to a pressure tube and left to dry. Once dried, the sample was treated at 95°C for 140 min with a chloroform:methanol:sulfuric acid solution (2:1.85:0.15 v/v) supplemented with 5 g/L of methyl benzoate as an internal standard. Following this procedure, 2 mL of water were added, and the mix was vortexed and left to settle. After settling the lower layer (organic phase) was extracted with the use of a syringe, filtered (0.45 μm PTFE filter) and transferred to a GC vial.

Gas chromatography with a flame ionization detector (GC-FID), a 30 m × 0.25 mm × 0.25 µm Zebron ZB-SemiVolatiles Capillary GC column capillary column (SGE Analytical Science) was used to analyse the PHA concentration. The method consisted of an initial temperature of 100°C for 3 min, followed by a ramp of 25°C/min to 200°C, followed by a second ramp of 30°C/min to 220°C, maintaining this temperature for 2 min. Helium was used as carrier gas. Standards were prepared from Methyl-(R)-3-hydroxybutyrate and Methyl-(R)-3-hydroxyvalerate (Sigma Aldrich) to create calibration curves.

#### 2.4.4 Determination of PHBV microstructure by NMR

Nuclear Magnetic Resonance (NMR) (B500 MHz Avance II+, Bunker), was used to determine the microstructure of the PHA produced by analysing the presence of HV and HB monomers and their distribution in the copolymer which can be identified by the position of the ^1^H and ^13^C present. The results allow calculation of the *D* value (Eq. (1)) which gives an idea of the relative proximity of HB*HV and HV*HB groups. A large *D* value corresponds to a block polymer structure while values close to 1 indicate a random polymer structure (Kamiya et al., 1989). The *D* value is given by:

$$D=\frac{F_{HBHB}\times F_{HVHV}}{F_{HBHV}\times F_{HVHB}}$$ (1)

Where F_HBHB_ represents the fraction of 3HB adjacent to 3HB monomer units, F_HBHV_ represents the fraction of 3HB adjacent to 3HV monomer units, F_HVHV_ represents the fraction of 3HV adjacent to 3HV monomer units, F_HVHB_ represents the fraction of 3HB adjacent to 3HV monomer units.

#### 2.4.5 Quantification of fatty acids with HPLC, yields

The supernatant from each OD sample was analysed to determine carbon substrate (VFA) content. Samples were passed through 0.45 µm Nylon filters before being transferred to HPLC vials. The C4:0 and C5:0 concentrations were quantified by High Performance Liquid Chromatography (HPLC) (Ultimate 300 Dionex HPLC system, Thermo Scientific) using an Aminex HPX-87H (Biorad) column and UV detector. The mobile phase used was 0.5 mM H_2_SO_4_. The operating temperature was 50°C, 1.0 mL/min flow rate and 220 nm UV wavelength. Pure valeric and butyric acid were used to prepare standards.

Product yield (Y_PHA/S_) and Biomass yield (Y_X/S_) were calculated with the following equations Y_PHA/S_ = [PHA _average_]/ΔS and Y_X/S_ = X _total_/ΔS. Where X represents the total biomass in g/L, = [PHA _average_] shows the average PHA concentration in g/L and ∆S the substrate consumed in g/L.

#### 2.4.6 Carbon consumption and feeding rate calculation

HPLC analysis results were used to obtain the fatty acid consumption rate which was calculated as the difference in VFA between two readings. The amount of VFA fed between samples was also taken into consideration. Substrate consumption was calculated as ΔS = [VFA]_feed_–[VFA]_outlet stream_ for each 24 h period.

The feed flow rate (mL h^-1^) was calculated as the product of reactor volume (mL) and dilution rate (h^-1^). The dilution rate was selected based on previous experiments as the expected specific growth rate for each 24 h period. The feed concentration (g mL^-1^) was determined based on the measured carbon consumption (g) and total volume fed over a 24 h period (mL).

## 3 Results and discussion

The results of two fully continuous bioreactor fermentations are presented, to prove the viability of a continuous mode of bioreactor operation for PHBV production in *Haloferax mediterranei* when fed a mix of C4:0/C5:0 volatile fatty acids (VFAs). Firstly, operating conditions, e.g., feeding rate, feed carbon concentration and steady state OD, are determined followed by discussion of continuous fermentation data and the analysis of the PHBV produced.

### 3.1 Design of continuous operating conditions

A continuous strategy, with feeding and broth removal, was designed to keep the cell density and carbon concentration constant inside the bioreactor. Two experiments were completed with a mix of C4:0/C5:0 VFAs at different media substrate concentrations; experiments A and B were performed at 0.1 M and 0.25 M carbon concentration respectively.

The natural PHBV production in *Haloferax mediterranei* is growth associated; maximum production occurs during the exponential growth phase (Koller et al., 2007). When looking to maximise productivity, elongating that exponential productive growth phase is of interest. When working in fed-batch and continuously fed fed-batch (CFFB) strategies, the maximum cell density observed was around OD 35 with exponential growth occulting in the range 8–35 OD (Ferre-Guell and Winterburn, 2019; Parroquin Gonzalez and Winterburn, 2022). Based on this a steady state OD between 10–12 was chosen for the continuous fermentations. These values fall at the beginning of the exponential phase which not only guarantees PHA production but also results in a relatively straight forward steady state at which to control and maintain the fermentation vs. a higher, late exponential stage, OD. Whilst operating at higher OD is possible it would require significantly greater dilution rates, achieved with high and difficult to control inlet and outlet flow rates.

**FIGURE 1:**
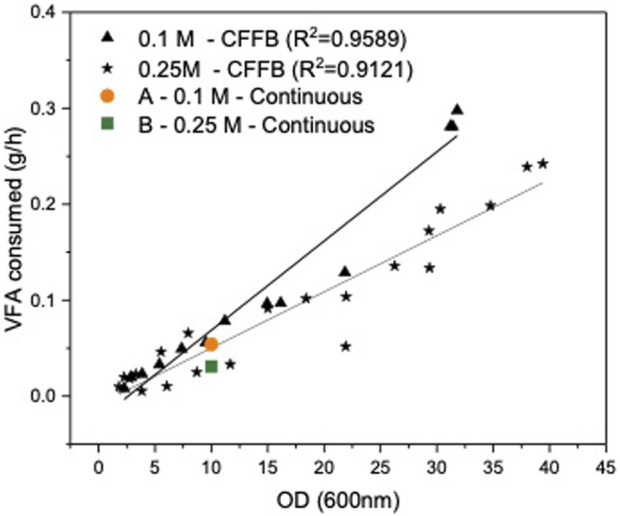
Volatile fatty acid consumption (g/L) plotted against cell density (OD). In black, results from the continuously fed fed-batch (CFFB) fermentations (Parroquin Gonzalez and Winterburn, 2022). Orange circle and green square consumption for experiments A (0.1 M) and B (0.25 M) respectively.

To establish a steady state cell density and carbon concentration inside of the reactor for the duration of the continuous fermentation, the outlet stream had to be set to match cell growth rate and carbon consumption rate. The inlet stream was set to match the outlet stream to maintain constant working volume in the reactor. The flowrates and feeding rate were initially calculated based on the growth rate in the CFFB fermentations averaging the same carbon concentration in the media. Both experiments were initially fed a C4:0/C5:0 (56:44 mol%) 0.3 M of carbon mix, same concentration as the CFFB experiments (Parroquin Gonzalez and Winterburn, 2022). However, with a continuous stream of cells leaving the reactor and hence limiting their natural exponential growth phase, cell growth and carbon consumption rates decreased when compared to CFFB fermentations and the outlet flow rate had to be adjusted; accordingly, experiments A and B had an outlet flow of 5.3 and 9.2 mL h^-1^ respectively. Once the outlet flow was stablished, the inlet flow had to be adjusted to keep the volume inside the reactor constant. The new consumption rate was calculated by analysing the carbon concentration change in the media over a 24 h period, samples were analysed by HPLC. A consumption rate of 54.1 and 30.9 mg h^-1^ were obtained for experiments A and B respectively. Research conducted in *Haloferax mediterranei* shows its preference for C4:0 over C5:0 during the growth phase (Ferre-Guell and Winterburn, 2019). Additionally, it has been documented that cells exhibit faster growth when the carbon concentration in the media is maintained at 0.25 M (Parroquin Gonzalez and Winterburn, 2022). As expected, B shows faster cell growth, as evidenced by the higher flow rate out (Table 1) which suggests that the cells are at a stage that further favors the consumption of C4:0 over C5:0. The carbon consumed is not only used by the cells for biomass growth but also for PHA production and formation of other fermentation products; experiment A shows slower growth with higher PHA production and a larger carbon consumption rate when compared to experiment B, a faster growing culture with less PHA production and lower carbon consumption. Figure 1 shows the consumption rate trends for the CFFB fermentation at increasing OD as well as the consumption rate values for experiments A and B. Knowing how much carbon had been consumed over that period as well as the required flowrate rate needed to keep the volume in the reactor at steady state, the final feeding concentrations for each experiment were calculated; experiments A and B where fed a media supplemented with 0.515 and 0.390 M of carbon respectively. The final design parameters used are presented in Table 1.

**TABLE 1:** Fermentation design parameters.

Exp	Media concentration (M)	Working Volume (L)	Steady state OD	Dilution rate (h^−1^)	Flowrate out (mL h^−1^)	Feeding concentration (M of Carbon)	VFA consumption rate (mg h^−1^)	C4:0 consumption rate (mg h^−1^)	C5:0 consumption rate (mg h^−1^)
*A*	0.1	1.2	10–12	0.0044	5.3	0.515	54.1	27.2	26.9
*B*	0.25			0.0076	9.2	0.390	30.9	22.3	8.6

### 3.2 Continuous fermentation results

**FIGURE 2:**
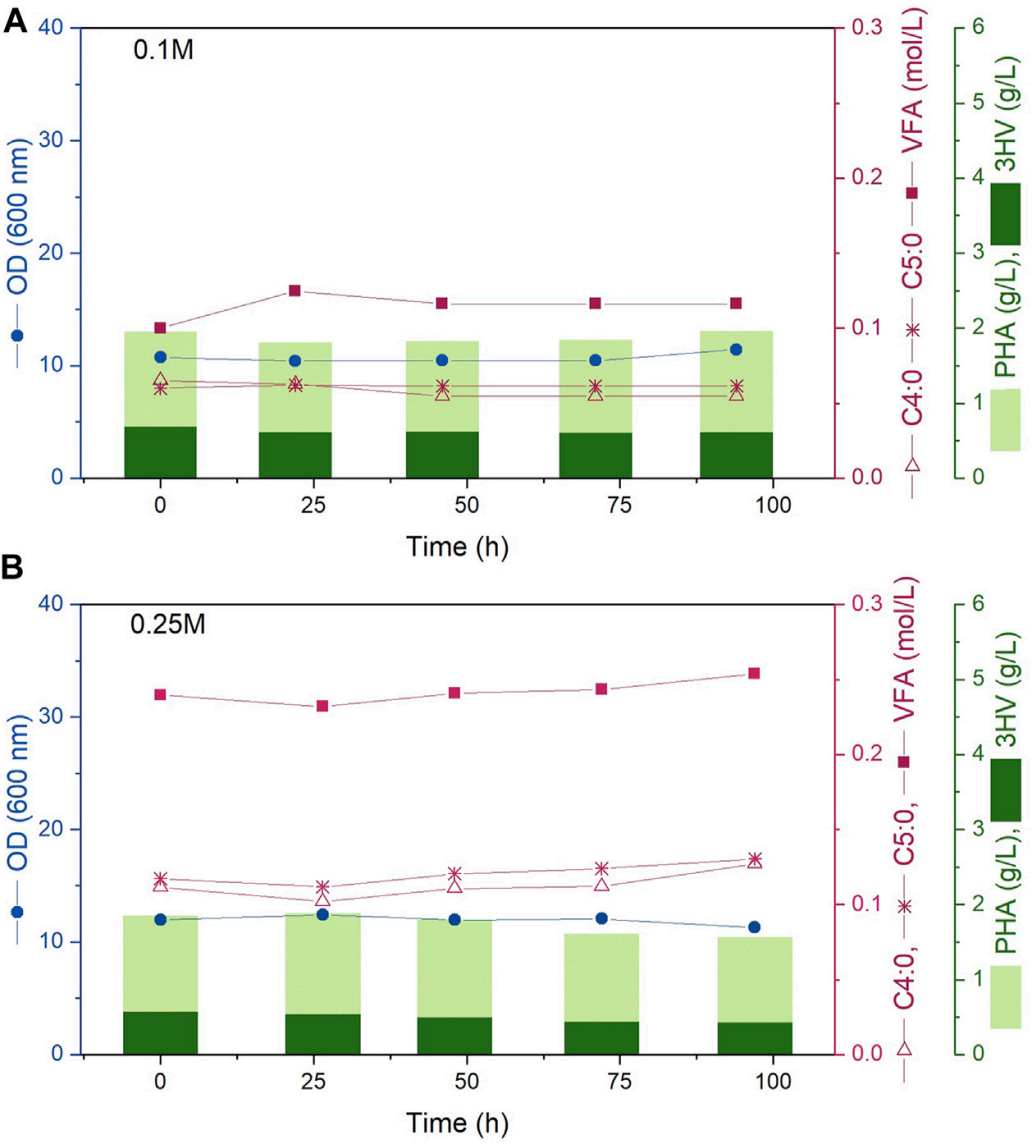
Bioreactor fermentation time course showing experiments **(A, B)** (top to bottom) corresponding to steady state carbon concentrations in the media of 0.1 and 0.25 M. The circles represent OD (600 nm), squares indicate VFA concentration (mol/L), empty triangles show C4:0 concentration (mol/L), asterisks represent C5:0 concentration (mol/L). The light bars represent the PHBV concentration while the dark bars represent the PHV concentration (g/L).

Results from two continuous fermentations are presented; experiments A and B were performed at steady state 0.1 M and 0.25 M carbon concentration in the media respectively. Both reactors were inoculated so that the initial cell density in the reactor would be within the desired 10–12 OD mark. Immediately following inoculation, the inlet and outlet streams were turned on and the continuous fermentation was initiated maintaining the working volume inside each bioreactor constant at 1.2 L. Both experiments were sampled every 24 h for 100 h; the data obtained for OD, VFA concentration in media, PHBV and 3HV concentration is presented in Figure 2. As desired, for both experiments A and B, each sample presented an OD value within the 10–12 mark. The carbon concentration in the media was successfully kept at 0.1 and 0.25 M of carbon concentration respectively through the 100 h analysed showing that the feeding concentrations and rates described in Table 1 were correctly calculated. In addition, both the concentration of the polymer and the 3HV fraction obtained remained constant over time. All these constant values demonstrate that steady state was successfully kept. Figure 2 shows steady state carbon concentration, cell density (OD) and polymer production remained constant for the duration of the continuous experiment. While the experiments presented in this work only lasted for 100 h, *Haloferax mediterranei* has been shown to be a stable culture for continuous processes with no genomic alterations observed after 3 months (Garcia Lillo and Rodriguez-Valera, 1990).

For the operations presented in this work, the continuous feed flow prevented salt deposits forming and salt accumulation was not observed in the tubing system, allowing for the feeding solution to include the required salts. This presents an additional advantage for the continuous operation granting greater control and assuring the fermentation is continuously provided the needed amount of micronutrients. While the high salinity might present an industrial scale up limitation due to potential for corrosion and salt build up, the experimental work presented in this paper shows that when there is a continuous flow of media salts do not accumulate, further careful material selection for bioreactor vessels and pipework can mitigate the corrosion risk when scaling up.

Overall, the results presented demonstrate that not only a continuous fermentation of *Haloferax mediterranei* is viable, but the PHBV produced is obtained continuously with constant concentration and 3HV fraction. This presents a huge industrial advantage as fermentations could be kept running for longer (at least 3 months), as opposed to being limited by the end of the exponential cell growth or by-products accumulation in batch fermentations, with product being harvested continuously whilst maintaining consistent material properties and quality. Continuous operation also minimises downtime, maximising the production possible from a given equipment asset.

### 3.3 PHA production and quantification in continuous fermentations

**FIGURE 3:**
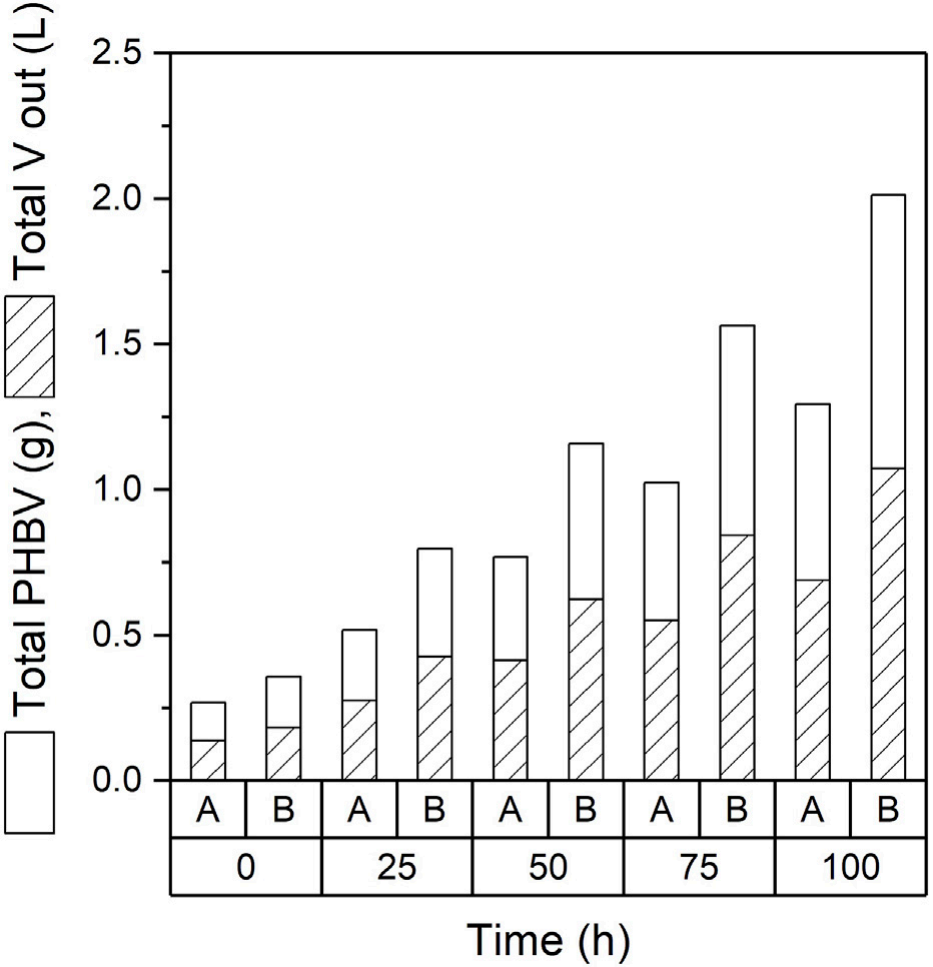
Total volume extracted, and polymer produced by continuous fermentations for experiments A (0.1 M) and B (0.25 M).

The main advantage of a continuous mode of operation is the ability of product to be harvested continuously. Figure 3 shows the total amount of PHBV and volume of broth obtained throughout the 100 h sampled. An average of 130 mL of broth containing 250 mg of PHBV were obtained daily from experiment A. Experiment B delivered an average of 220 mL containing 376 mg of PHBV. By the end of the continuous production time more than 600 mL had been extracted from experiment A with a total of 1.2 g of PHBV produced while 1,100 mL and 2 g of PHBV were produced in experiment B (Figure 3).

Three different productivities were calculated for both experiments to characterise the fermentation and be able to compare them with the results previously obtained for CFFB operations (Table 2). Overall productivities of 9.6 and 17.2 mg L^-1^ h^-1^ for experiments A and B respectively were obtained. A productivity normalised by OD of 0.87 and 1.43 mg L^-1^ h^-1^ OD^−1^ was calculated for experiments A and B respectively. Finally, a steady state productivity of 77.5 and 71.3 mg L^-1^ h^-1^ for A and B respectively was obtained.

**TABLE 2:** Parameters for the PHBV produced in *Haloferax mediterranei* grown in different VFA constant concentrations and operation conditions.

	Operation mode	Carbon concentration (M)	Time (h)	PHBV (g L^−1^)	Total PHBV produced (g)	Overall Productivity (mg L^−1^ h^−1^)	Maximum OD	Productivity normalised by OD (mg L^−1^ h^−1^ OD^−1^)	Steady state productivity (mg L^−1^ h^−1^)	Steady state production rate (mg h^−1^)	Y_X/S_ (g g^−1^)	Y_PHA_ (g g^−1^)
*A*	Continuous	0.1	104	1.86	1.2	9.6	11^b^	0.87	77.5	9.9	0.07	0.18
*B*	Continuous	0.25	97	1.71	2.0	17.2	12^b^	1.43	71.3	15.8	0.32	0.51
*A^a^*	Fed-batch	0.1	260	5.27	9.5	10.0	34	0.29	N/A	N/A	0.29	0.73
*B^a^*	Fed-batch	0.25	383	4.73	9.4	12.8	34	0.38	N/A	N/A	0.29	0.63

^a^ Values for continuously fed fed-batch fermentations were taken from Parroquin Gonzalez and Winterburn (2022).

^b^ Average OD values at steady state.

The overall productivity for CFFB fermentations was 10.0 and 12.8 mg L^-1^ h^-1^ when fed 0.1 and 0.25 M of carbon respectively (Parroquin Gonzalez and Winterburn, 2022). For the experiments at 0.1 M VFA the overall productivity calculated is numerically equal for CFFB and continuous fermentations (Table 2) while in the 0.25 M experiments there is a 34% increase in overall productivity from 12.8 mg L^-1^ h^-1^ in CFFB to 17.2 mg L^-1^ h^-1^ in the continuous process. However, while these values are mathematically calculated in the same manner accounting for the total polymer mass produced in the total working volume during the total fermentation time, the significance is of the value is different; the CFFB total fermentation time goes from the start to the actual end of the fermentation life (fermentation reaching stationary phase) while the continuous total time only accounts for the 100 h tested while the fermentation could have continued for a much longer time at least tripling the CFFB time. In addition, the polymer produced in the continuous fermentations was made by a lower total cell count with the total cell density staying between 10–12; in the CFFB the amount of polymer produced increases proportionately with the cell density going up to 34 OD.

To be able to compare productivities, the overall productivity is normalised by the maximum OD obtained. When comparing these values, the productivity normalised by OD increased from 0.29 to 0.87 mg L^-1^ h^-1^ OD^−1^ (331%) and from 0.38 to 1.43 mg L^-1^ h^-1^ OD^−1^ (453%) for 0.1 and 0.25 M carbon concentration respectively for CFFB and continuous fermentation (Table 2). In this work, only one OD value was tested in continuous operation, higher cell densities could be tested in the future to further improve the overall productivity.

Steady state productivity and production rates were also calculated and are presented in Table 2. These values correspond to the 24 h period between samples and can be useful when comparing fermentations at different working volumes. Steady state productivities of 77.5 and 71.3 mg L^-1^ h^-1^ were obtained for experiments A and B respectively. One of the main features of continuous fermentations is the constant product delivery; steady state production rates of 9.9 and 15.8 mg h^-1^ were obtained for experiments A and B respectively.

Biomass yields of 0.07 and 0.32 (g g^-1^) were obtained for experiments A and B respectively. As for the PHA yield, values of 0.18 and 0.51 (g g^-1^) were obtained for experiments A and B respectively (Table 2). In the CFFB experiments conducted at the same carbon concentration the biomass yields obtained were of 0.29 (g g^-1^) for both 0.1 and 0.25 M fermentations and PHA yields of 0.73 and 0.63 (g g^-1^) were calculated for 0.1 and 0.25 M fermentations respectively (Parroquin Gonzalez and Winterburn, 2022). Experiment A did not show an improvement in terms of yield while experiment B presented an improved biomass yield and maintained a similar value for PHA yield. It is important to know than in CFFB cells were allowed to grow all the way to stationary phase producing polymer at higher cell density. Engineering the *Haloferax mediterranei* cells with an additional phaCAB operon and exploring higher cell density continuous fermentations can be ways to further increase the yields. However, even with the lower yields observed the greater productivity normalised by OD, continuous production and elimination down time associated with batch changeover means the continuous process is a more attractive mode of operation compared to fed-batch.

An in-depth techno-economical assessment for PHBV production *via* fermentation has been published (Policastro et al., 2021). In this study the productivity and 3HV fraction of different PHBV processes are compared. It is known that PHBV copolymers with 30–60 mol% 3HV composition are elastic and soft which makes them desirable over other polymeric compositions that are less elastic and brittle (Sudesh et al., 2000; Anjum et al., 2016). Furthermore, 3HV fractions higher than 30 mol% have a lower melting temperature, increasing the gap between melting and degradation temperatures facilitating their moulding into desired goods (Lauzier et al., 1992; Khanna and Srivastava, 2005). However, 3HV factions in *Haloferax mediterranei* are limited by precursor toxicity with higher than 15% are difficult to achieve; 3HV fractions higher than 40 mol% rarely being reported (Fernandez-Castillo et al., 1986; Chen et al., 2006). Overall productivities associated with these processes range between 9 and 360 mg L^-1^ h^-1^ with the highest values obtained when glucose or starch are used as carbon sources (Don et al., 2006; Hermann-Krauss et al., 2013). While said productivities are numerically larger than those presented here, when using a mix of C4:0 and C5:0 VFAs as carbon source it is possible to control the 3HV fraction and obtain compositions as high as 99.5 mol% without toxicity limitation (Ferre-Guell and Winterburn, 2018). 3HV plays an important role in the market price with higher fractions being more desirable. Furthermore, most processes use a batch operation mode hence process productivity is limited by fermentation cycles with time between batches having a large impact in overall production and product pricing. Having steady state PHBV production capacity makes the process more efficient despite the relatively low productivities. Whilst further process development is still required to further increase productivity in order for this system to become industrially viable, overall the productivities reported here confirm that a continuous fermentation in which high 3HV fractions are achievable has a greater potential than the previously conducted batch fermentations.

With higher productivities, production rate and yields obtained for experiment B, it is shown that a higher carbon concentration in the media is recommended, whilst keeping in mind that concentrations close and above to 0.4 M will become toxic for *Haloferax mediterranei* (Parroquin Gonzalez and Winterburn, 2022).

### 3.4 Polymer structure

**FIGURE 4:**
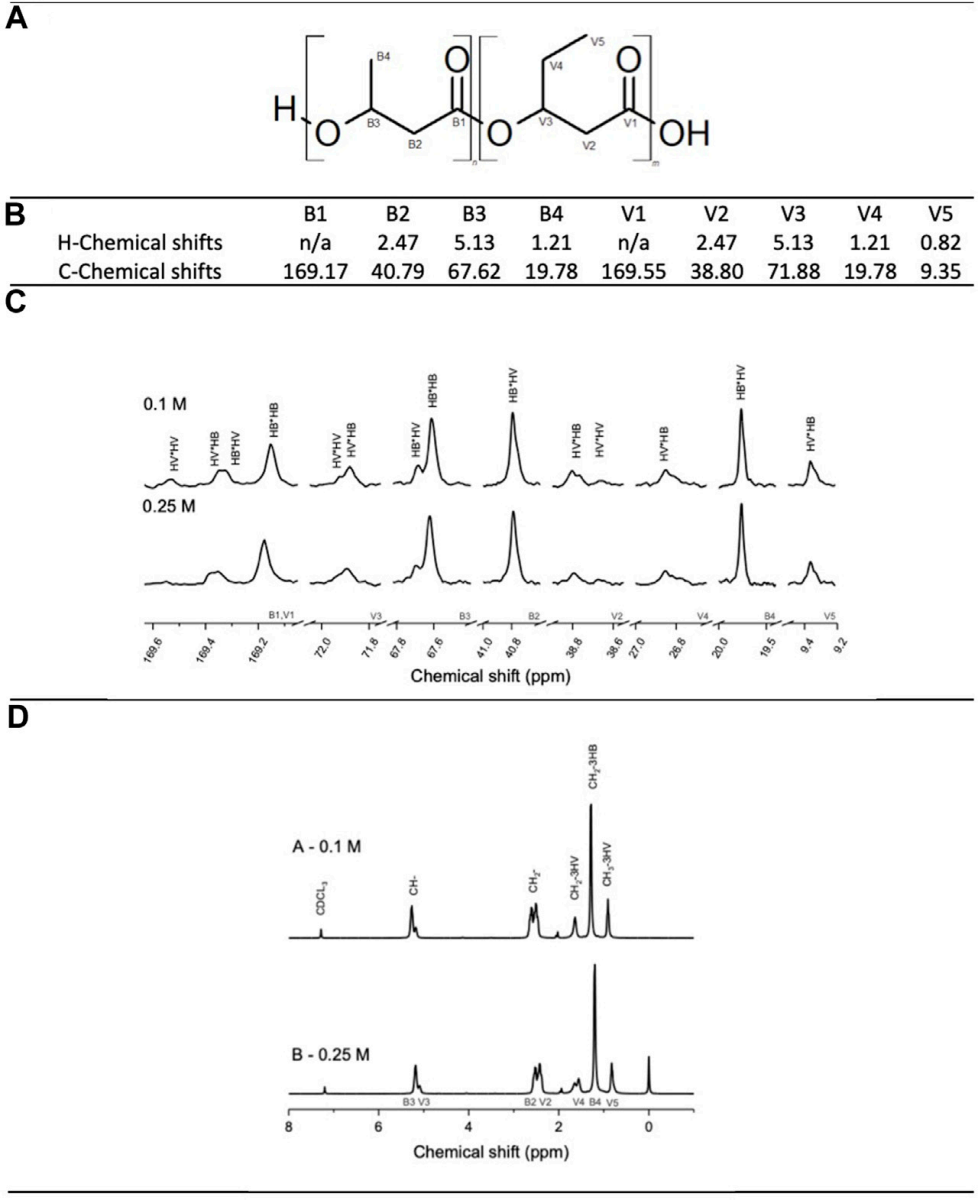
Top to bottom: **(A)** PHBV structure highlighting each carbon of the chain: B_i_ and V_i_ carbons correspond to PHB and PHV monomers respectively. **(B)** Characteristic chemical shifts for each carbon and proton in the PHBV molecule. **(C)** C-NMR and **(D)** H-NMR spectrums of experiments A and B.

Nuclear magnetic resonance (NMR) was performed to determine the copolymer structure and to confirm the presence of PHB and PHV in the copolymer. Figure 4 shows the chemical shifts obtained in the ^13^C-NMR and H-NMR spectrums. With the integrated value of the peaks corresponding to HB-HB, HB-HV, HV-HV and HV-HB neighbouring units, the *D* values were calculated, see Table 3. With a D values close to 1; 1.12 and 1.02 for experiments A and B respectively, a random polymeric structure is suggested for both experiments A and B (Žagar et al., 2006) (Table 3). Given that C4:0 and C5:0 were continuously co-fed the assimilation of the substrate and addition of PHB and PHV monomers into the PHBV polymeric chain would occur concurrently creating a chain with a random structure. A random allocation guarantees a well-mixed polymer with enhanced mechanical properties; with PHB being a more brittle and less elastic material it is not desirable to have long sections of it in the polymer chain (Anjum et al., 2016).

**TABLE 3:** D values for polymer structure characterisation.

Experiment	D Value	Polymer composition
*A—0.1 M*	1.12	Random
*B—0.25 M*	1.02	Random

## 4 Conclusion

This work shows for the first time that continuous PHBV production in *Haloferax mediterranei* when fed volatile fatty acids is viable. Controlling and maintaining a steady state carbon concentration and cell density in the reactor resulted in a significant productivity normalised by OD improvement with respect to previous fed-batch strategies tested. When performing fermentations following this regime, productivity normalised by OD increased from 0.29 to 0.38 mg L^-1^ h^-1^ in continuously fed-fed batch fermentations to 0.87 and 1.43 mg L^-1^ h^-1^ when continuously operated for 0.1 and 0.25 M carbon concertation in the media respectively. Production rates of 9.9 and 15.8 mg h^-1^ for 0.1 and 0.25 M carbon concertation respectively were achieved, showing continuous product delivery is possible. Presenting the first continuous fermentation for PHBV production in *Haloferax mediterranei*, this paper offers a step towards the wider industrialisation of PHBV production by fermentation, offering a process that is relatively straight forward to operate and produces a relatively high product concentration.

## Data Availability

The raw data supporting the conclusions of this article will be made available by the authors, without undue reservation.
